# *Clubiona jiugong* sp. nov., the fifth species of *C. zilla*-group from China (Araneae: Clubionidae)

**DOI:** 10.3897/BDJ.9.e66260

**Published:** 2021-04-29

**Authors:** Zuxian Zeng, Da Wang, Wanjuan Song, Hao Yu, Yang Zhong

**Affiliations:** 1 School of Biological Sciences, Guizhou Education University, Guiyang, China School of Biological Sciences, Guizhou Education University Guiyang China; 2 Hubei Key Laboratory of Radiation Chemistry and Functional Materials, School of Nuclear Technology and Chemistry & Biology, Hubei University of Science and Technology, Xianning, China Hubei Key Laboratory of Radiation Chemistry and Functional Materials, School of Nuclear Technology and Chemistry & Biology, Hubei University of Science and Technology Xianning China

**Keywords:** new species, morphology, diagnosis, DNA barcode, taxonomy

## Abstract

**Background:**

The *Clubiona zilla*-group is a relatively small species group, distributed exclusively in East Asia, with only three species clearly documented so far.

**New information:**

*Clubiona hooda* Dong & Zhang, 2016, which was previously placed in the *C. trivialis*-group, is assigned to the *C. zilla*-group in the present paper. A new spider of the *C. zilla*-group from Jiugong Mountain in China is described under the name of *C. jiugong* sp. nov. Detailed descriptions and photographs of the new species are provided.

## Introduction

The female of *Clubiona zilla* Dönitz & Strand, 1906 was reported by Ono (1986) for the first time. In the same paper, a new and peculiar species group, *C. zilla*-group, was established to accommodate *C. zilla*. The *C. zilla*-group was redeﬁned by Mikhailov (1995a) and Mikhailov (1995b) and was raised to genus *Anaclubiona* by Ono (2010). Later, the elevation of *Anaclubiona* was rejected by Mikhailov (2012) and the genus was suppressed and reverted to the *zilla*-group. The genus *Anaclubiona* Ono, 2010 is currently considered as a junior synonym of *Clubiona* by World Spider Catalog (2021).

According to Mikhailov (1995a), Mikhailov (1995b), Ono (1986), Ono (2010), the *zilla*-group can be easily recognised by its tiny body (with body length 1.8–3.6 mm), in association with the characteristic genital organs. The male palp has a developed and occasionally branched embolic apophysis. The female epigyne has a pair of guide pockets (or hoods) or a transverse hood near the copulatory openings. The *zilla*-group is a relatively small taxon, with only three species having been clearly documented: *C. zilla* widespread in Japan, *C. minima* (Ono, 2010) endemic to Honshu in Japan, *C. tanikawai* Ono, 1989 from Ryukyu Is. in Japan and Hunan and Taiwan in China. *C. hooda* Dong & Zhang, 2016 was assigned to the *trivialis*-group in the original publication (Dong and Zhang 2016), although it exhibits typical *zilla*-group features.

**Figure 1.**
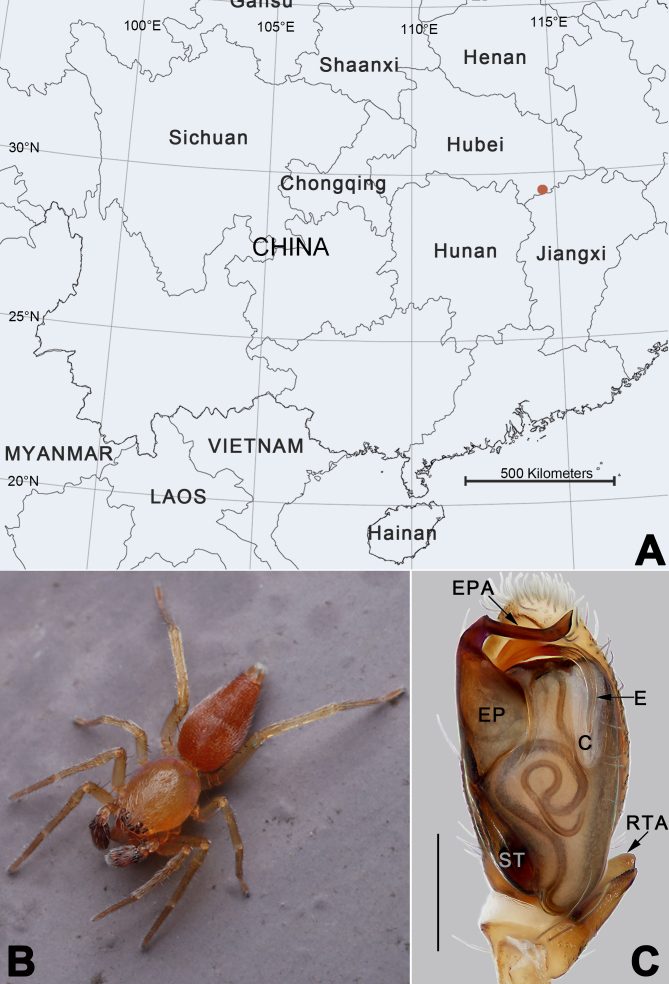
*Clubiona jiugong* sp. nov. **A.** Distribution record (red circles); **B.** Male holotype; **C.** Male left palp of the holotype, ventral view. Abbreviations: C = conductor; E = embolus; EP = embolar part; EPA = embolar part apophysis; RTA = retrolateral tibial apophysis; ST = subtegulum. Scale bar: 0.1 mm (C). The photograph of the living spider was provided by Qianle Lu (Shenzhen, Guangdong).

While examining spiders collected from Jiugong Mountain, Hubei Province, China (Fig. 1A), we found pairs of *Clubiona* specimens in the same location, which are with similar habitus, markings, leg spination and other characters (Fig. 1B and Figs. 3E–H) and both sexes possess certain characters associated with the *zilla*-group. Therefore, it is very likely they are the opposite sexes of the same species. Based on that, as well as the DNA barcoding data, we matched the female and male together. This species is new to science and is described under the name of *C. jiugong* sp. nov. The aim of the current paper is to describe the new species, providing detailed morphological descriptions and illustrations.

## Materials and methods

Specimens in this study were collected by hand collecting from leaf-litter in Mt. Jiugong, Hubei. Spiders were fixed and preserved in 95% ethanol. Specimens were examined with an Olympus SZX7 stereomicroscope, details being studied with an Olympus CX41 compound microscope. Female epigynes and male palps were examined and illustrated after being dissected. Epigynes were removed and cleared in warm lactic acid before illustration. The vulva was also imaged after being embedded in Arabic gum. Photos were made with a Cannon EOS70D digital camera mounted on an Olympus CX41 compound microscope. The digital images were taken and assembled using Helifocus 3.10.3 software package (Khmelik et al. 2006). The distribution map was generated with ArcView GIS 3.2 (ESRI 2002).

A DNA barcode was also obtained for the species matching. A partial fragment of the mitochondrial cytochrome oxidase subunit I (CO1) gene was amplified and sequenced for two specimens, using the primers LCOI1490 (5’-GGTCAACAAATCATAAAGATATTG-3’) and HCOI2198 (5’-TAAACTTCAGGGTGACCAAAAAAT-3’). For additional information on extraction, amplification and sequencing procedures, see Malumbres-Olarte and Vink (2012).

All measurements were obtained using an Olympus SZX7 stereomicroscope and given in millimetres. Eye diameters are taken at the widest point. The total body length does not include chelicerae or spinnerets length. Leg lengths are given as total length (femur, patella, tibia + metatarsus, tarsus). Most of the terminologies used in text and figure legends follow Dong and Zhang (2016).

All specimens are deposited Museum of Guizhou Education University, Guiyang, Guizhou, China (MGEU, curator Hao Yu).

## Taxon treatments

### Clubiona jiugong

Yu & Zhong sp. n.

9B74C4BF-3DDC-5DB0-98D9-7D1EDCFDB514

36f4d405-32d2-4119-97b6-9c3758c20f22

#### Materials

- **Type status:** Holotype. **Occurrence:** recordedBy: Qianle Lu; individualID: YHCLU0274; individualCount: 1; sex: male; lifeStage: adult; behavior: foraging; preparations: whole animal (EtOH); associatedSequences: GenBank: MZ020606; **Taxon:** order: Araneae; family: Clubionidae; genus: Clubiona; specificEpithet: *jiugong*; scientificNameAuthorship: Yu & Zhong; **Location:** continent: Asian; country: China; countryCode: CHN; stateProvince: Hubei; county: Tongshan; locality: Jiugongshan Nature Reserve; decimalLatitude: 29.39; decimalLongitude: 114.65; **Identification:** identifiedBy: Hao Yu; dateIdentified: 2020-07; **Event:** samplingProtocol: by hand; samplingEffort: 10 km by foot; year: 2020; month: 7; day: 3; **Record Level:** institutionCode: MGEU; basisOfRecord: Preserved Specimen
- **Type status:** Holotype. **Occurrence:** recordedBy: Qianle Lu; individualID: YHCLU0275; individualCount: 1; sex: female; lifeStage: adult; behavior: foraging; preparations: whole animal (EtOH); associatedSequences: GenBank: MZ020605; **Taxon:** order: Araneae; family: Clubionidae; genus: Clubiona; specificEpithet: *jiugong*; scientificNameAuthorship: Yu & Zhong; **Location:** continent: Asian; country: China; countryCode: CHN; stateProvince: Hubei; county: Tongshan; locality: Jiugongshan Nature Reserve; decimalLatitude: 29.39; decimalLongitude: 114.65; **Identification:** identifiedBy: Hao Yu; dateIdentified: 2020-07; **Event:** samplingProtocol: by hand; samplingEffort: 10 km by foot; year: 2020; month: 7; day: 4; **Record Level:** institutionCode: MGEU; basisOfRecord: Preserved Specimen

#### Description

**Male** (Fig. 3E and F). Dimensions in mm. Total length 2.60; carapace 1.33 long, 1.02 wide; abdomen 1.27 long, 0.75 wide.

Colour of the living holotype male was dark brown with red brown abdomen (Fig. 1B). *Carapace* yellowish-brown in ethanol (Fig. 3E and F), without a distinct pattern. Fovea red. In dorsal view, anterior eye row (AER) slightly recurved, posterior eye row (PER) almost straight, PER wider than AER. Eye sizes and interdistances (mm): anterior median eyes (AME) 0.08, anterior lateral eyes (ALE) 0.09, posterior median eyes (PME) 0.08, posterior lateral eyes (PLE) 0.07; distance between AMEs (AME–AME) 0.03, distance between AME and ALE (AME–ALE) 0.04, distance between PMEs (PME–PME) 0.16, distance between PME and PLE (PME–PLE) 0.06. Length of median ocular quadrangle (MOQ) 0.18, MOQ anterior width 0.18, MOQ posterior width 0.29. *Chelicerae* coloured as carapace, with 5 teeth on promargin and 3 on retromargin. Labium and endites yellowish-brown. Sternum 0.70 long, 0.42 wide.

Abdomen red in ethanol (Fig. 3E and F), elongate-oval, dorsum centrally with a lengthwise reticular pattern, reaching 2/5^th^ of abdomen length, posteriorly with a fuzzy pattern represented by numerous horizontal stripes or blotches; ventre reddish-brown; spinnerets light brown.

Legs uniformly yellowish-brown in ethanol (Fig. 3E and F). Leg length (mm): I 2.62 (0.80, 1.05, 0.52, 0.25), II 2.84 (0.80, 1.30, 0.49, 0.26), III 2.27 (0.75, 0.78, 0.48, 0.25), IV 3.37 (1.11, 1.27, 0.98, 0.30).

**Figure 2.**
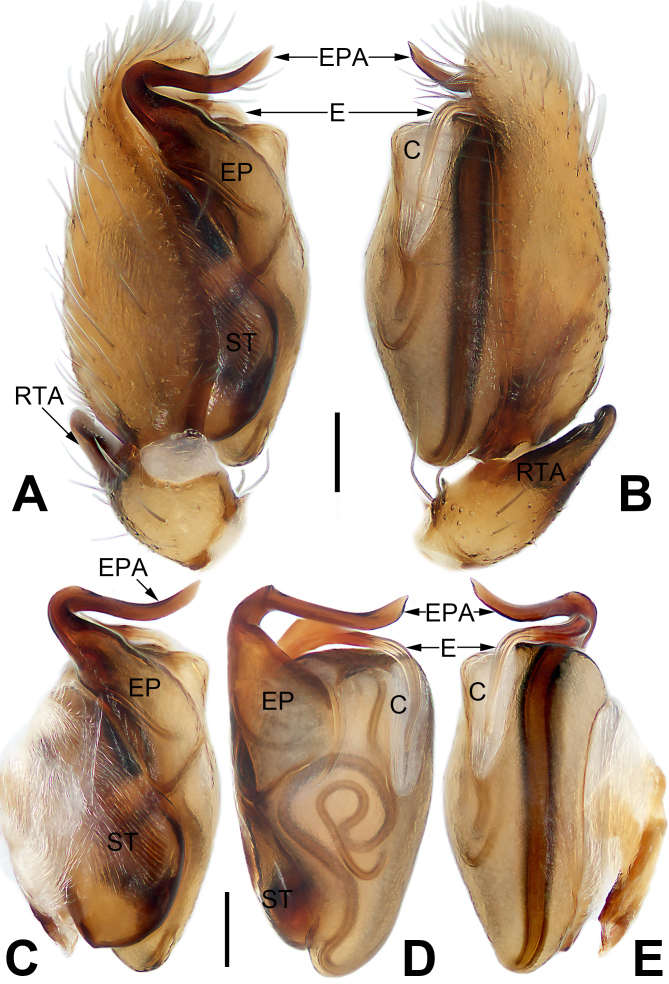
Male left palp of the holotype of *Clubiona jiugong* sp. nov. **A.** Prolateral view; **B.** Rretrolateral view; **C.** Bulb, prolateral view; **D.** Bulb, ventral view; **E.** Bulb, retrolateral view. Abbreviations: C = conductor; E = embolus; EP = embolar part; EPA = embolar part apophysis; RTA = retrolateral tibial apophysis; ST = subtegulum. Scale bars: 0.1 mm (equal for **A** and **B**, equal for **C–E**).

Palp (Fig. 1C and Fig. 2A–E). Femur and patella unmodified. Tibia short, with single retrolateral apophysis; retrolateral tibial apophysis (RTA) broad, triangular, distally bifurcate in retrolateral view, both tips blunt. Tegulum oval and relativlely flat, ca. twice longer than wide, sperm duct distinct and sinuous; subtegulum (ST) large, located prolaterally. Embolar part (EP) represented by a wide and flat sclerite, situated prolaterally on the tegulum; embolar part apophysis (EPA) strong, slender and long, about as long as tegulum width, shaped like a dagger, originating on the prolateral flank (approximately 11 o’clock on tegulum), transversally curved to the retrolateral side. Embolus (E) inserted at approximately ten o’clock on tegulum, slender and flagelliform, angled across tegular tip, stretched proximally along membranous conductor, tip extending to one-third of tegulum. Conductor (C) area relatively small, approximately two-fifths the length of tegulum.

**Figure 3.**
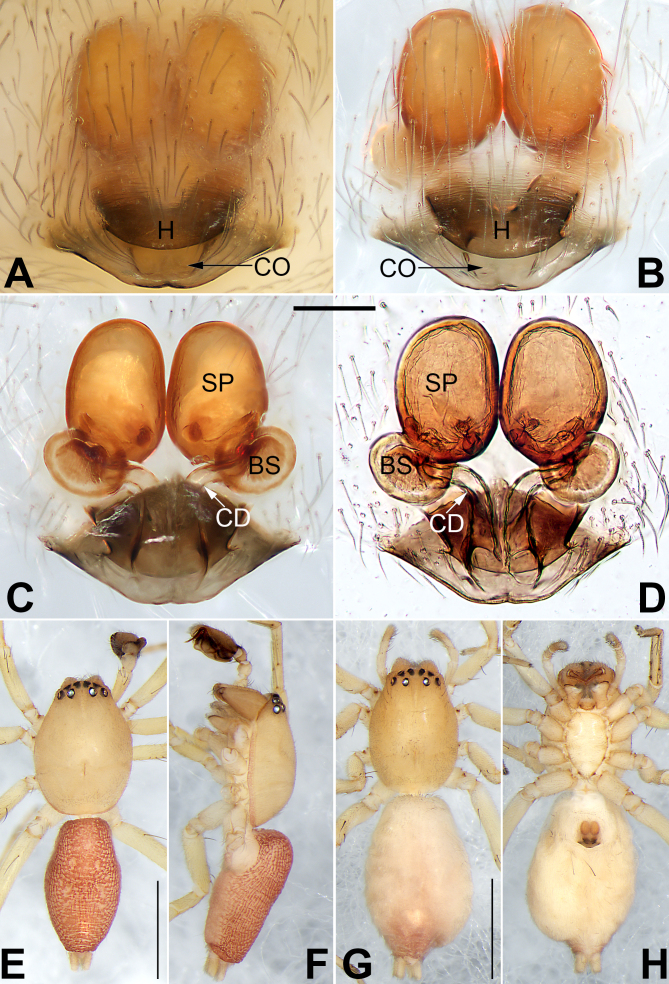
*Clubiona jiugong* sp. nov., female paratype and male holotype. **A.** Intact epigyne, ventral view; **B.** Cleared epigyne, ventral view; **C.** Cleared vulva, dorsal view; **D.** Vulva, cleared and embedded in Arabic gum, dorsal view; **E.** Male habitus, dorsal view; **F.** Male habitus, lateral view; **G.** Female habitus, dorsal view; **H.** Female habitus, ventral view. Abbreviations: BS = bursa; CD = copulatory duct; CO = copulatory opening; H = hood; SP = spermatheca. Scale bars: 0.1 mm (equal for **A–D**); 1 mm (equal for **E and F**, equal for **G and H**).

**Female** (Fig. 3G and H. Dimensions in mm) Total length 3.64; carapace 1.61 long, 1.14 wide; abdomen 2.03 long, 1.35 wide. Eye sizes and interdistances: AME 0.09, ALE 0.19, PME 0.07, PLE 0.07, AME–AME 0.05, AME–ALE 0.04, PME–PME 0.19, PME–PLE 0.09. MOQL 0.25, MOQA 0.23, MOQP 0.36. Sternum 0.87 long, 0.49 wide. Measurements of legs: I 2.65 (0.77, 1.10, 0.53, 0.25), II 2.79 (0.84, 1.15, 0.58, 0.22), III 2.52 (0.66, 0.98, 0.65, 0.23), IV 3.81 (1.25, 1.26, 0.94, 0.35). General characters as in female, but slightly larger in size and lighter in colour.

Epigyne (Fig. 3A–D). Epigynal plate distinctly longer than wide, anterior and lateral margin not delimited, posterior margin rebordered, heavily sclerotised and convex; spermathecae (SP) clearly visible through the tegument in ventral view. Two copulatory openings (CO) large, partly fused, situated at medial portion of epigynal plate posterior margin, anteriorly hidden by a hood. Hood (H) wider than 1/2 of epigyne width, heavily sclerotised, V or U-shaped. Hyaline copulatory ducts (CD) thin, ascending in parallel, the proximal half close together, the distal half widely separated and gently curved towards the bursae. Both spermathecae (SP) and bursae (BS) with smooth surfaces, the former anteriad and distinctly larger than the latter. Spermatheca shaped like a chicken egg, inside pigmented and sclerotised, the two spermathecae closely spaced. Bursae globular, separated by ca. 1.2 diameters.

**DNA barcode**

5'CTTGATCTGCTATAGCAGGAACAGCTATAAGTGTTATAATTCGTATAGAATTAGGACAATCTGGAACATTTTTAGGAGATGATCATTTATATAATGTAGTAGTTACAGCTCATGCTTTTGTTATAATTTTTTTTATAGTAATACCTATTTTAATTGGAGGTTTTGGAAATTGAATAATTCCTATGATATTAGGAGCAGCTGATATAGCTTTTCCTCGTATAAATAATTTAAGTTTTTGATTATTACCTCCTTCGTTATTTATATTATTTATATCTTCTATAGCTGAAATAGGTGTGGGAGCAGGGTGAACTATTTATCCTCCTCTTGCATCTAGTATAGGTCATACAGGAAGAGCTATAGATTTTGCTATTTTTTCGTTACATCTAGCTGGAGCTTCTTCTATTATAGGGGCTGTAAATTTTATTACTACTATTATTAATATACGATATATTGGGATGAGAATAGAAAAAGTTCCATTATTTGTTTGGTCTGTTATAATTACTGCAGTACTCTTATTATTATCATTACCTGTATTAGCAGGTGCTATTACTATATTATTGACTGATCGAAATTTTAATACATCTTTTTTTGATCCAGCTGGAGGGGGAGATCCTATTTTATTTCAGCATTTATTTTGATTTTTTGG3' (holotype, YHCLU0274; GenBank: MZ020606)

**DNA barcode**

5'TTTGATCTGCTATAGTAGGAACAGCTATAAGTGTTATAATTCGTATAGAATTGGGACAATCTGGAACATTTTTAGGAGATGATCATTTATATAATGTAGTAGTTACAGCTCATGCTTTTGTTATAATTTTTTTTATAGTAATACCAATTTTAATTGGAGGTTTTGGAAATTGAATAATTCCTATGATATTAGGAGCAGCTGATATAGCTTTTCCTCGTATAAATAATTTAAGTTTTTGATTATTACCCCCTTCGTTATTTATATTATTTATATCTTCTATAGCTGAAATAGGTGTGGGAGCAGGGTGAACTATTTATCCTCCTCTTGCATCTAGTATAGGTCATACAGGAAGAGCTATAGATTTTGCTATTTTTTCGTTACATCTAGCTGGAGCTTCTTCTATTATAGGGGCTGTAAATTTTATTACTACTATTATTAATATACGATATATTGGGATGAGAATAGAAAAAGTTCCATTATTTGTTTGGTCTATTATAATTACTGCAGTACTCTTATTATTATCATTACCTGTATTAGCAGGTGCTATTACTATATTATTGACTGATCGAAATTTTAATACATCTTTTTTTGACCCAGCTGGAGGAGGAGATCCTATTTTATTTCAGCATTTATTTTGATTTTTTGG3' (paratype, YHCLU0275; GenBank: MZ020605)

#### Diagnosis

*Clubiona jiugong* sp. nov. resembles the other *zilla*-group species by the similar habitus (tiny body with length not exceeding 4 mm), but is consistently separable by its genitalia. Male of the new species resembles that of *C. hooda* (Dong and Zhang 2016: 7, figures 5–7 and 10–12) in having a dagger-shaped EPA and a flagelliform embolus, but can be recognised by the RTA distally bifurcate (Fig. 1C) (vs. RTA not branched in *C. hooda*) and by the EPA originating from the prolateral portion of the tegulum, pointed to the retrolateral side (Fig. 1C, Fig. 2A and C–E) (vs. EPA originating retrolaterally and curved to the prolateral side). Females of *C. jiugong* sp. nov. can be easily distinguished from other members of the *C. zilla*-group, with the exception of *C. zilla* (Ono 1986: 119, figures 6–8) by the hood represented by a transverse sclerotised plate (hoods represented by pairs of guide pockets in all other *zilla*-group species) and differ from *C. zilla* by: (1) copulatory openings closely spaced and partly fused, situated at the medial portion of epigynal plate posterior margin (Fig. 3A and B) (vs. copulatory openings well separated by ca. 0.8 diameters, situated basolaterally in *C. zilla*); (2) the proximal half of copulatory ducts close together (Fig. 3A and B) (vs. the proximal half of copulatory ducts well separated by more than 4 diameters); (3) spermatheca oval and large, its diameter nearly 1/2 of epigynal width (Fig. 3C and D) (vs. spermatheca globular and small, its diameter slightly less than 1/5 of epigynal width).

#### Etymology

The species name is derived from the name of the type locality; noun in apposition.

#### Distribution

Known from the Mt. Jiugong, Hubei Province, China (Fig. 1A).

#### Biology

The holotype of *C. jiugong* sp. nov. was obtained from foliage in a bush close to a mountain road in the core zone of Jiugong mountain range.

## Discussion

The *zilla*-group morphologically is very similar to the *trivialis*-group. According to previous publications (Dondale and Redner 1982, Dong and Zhang 2016, Mikhailov 1990, Mikhailov 1995b, Ono 2010, Ono 1986, Yu and Li 2019), the two species groups share similar male palp: the retrolateral tibial apophysis simple, erect, not branched; the embolus arched around or angled across the distal end of tegulum; the conductor groove-like, membranous, fused to tegulum, located distally on retrolateral side of tegulum; the sperm duct meandering and distinct. That is perhaps the reason why *C. hooda* was placed in the *trivialis*-group in Dong and Zhang (2016). However, the *C. zilla*-group can be distinguished from the *C. trivialis*-group by the following combination of genitalic characters: for males, palp with a developed embolic apophysis (Dong and Zhang 2016: figures 5, 7, 10 and 12; Fig. 1C and Fig. 2A–E) lacking in *trivialis*-group; for females, presence of hood (or guide pockets) near the copulatory openings in female (Dong and Zhang 2016: figures 3 and 8; Fig. 3A and B) (vs. absent). The two groups also can be separated by their different sizes: *C. zilla*-group species with tiny bodies (less than 3.6 mm), *C. trivialis*-group is median sized clubionids (usually larger than 5 mm). Although we have not examined the types of *C. hooda*, the species was well described with high-quality illustrations: developed embolar part apophysis in male and the paired epigynal hoods in female (Dong and Zhang 2016: 7, figures 1–12), leave no doubts that our transfer is correct.

The *zilla*-group presents a distinct set of characters, is one of the most distinct groups of the genus *Clubiona* sensu lato and has been considered as putatively monophyletic (Mikhailov 1995a, Mikhailov 1995b, Ono 1986, Ono 2010). The group may be further separated from the genus *Clubiona* sensu lato and may be resurrected to genus level.

The genus *Clubiona* is very diverse and, so far, more than 500 valid species have been described. Before splitting such a genus, all current species should be considered, which certainly requires a large-scale study (i.e. a worldwide phylogenetic revision). The present study follows World Spider Catalog (2021) in regarding *Anaclubiona* as a synonym of *Clubiona*, rather than resurrecting the generic status of the *zilla*-group. Consequently, we temporarily place the new species in *Clubiona* sensu lato and assign them to *C. zilla*-group.

## Supplementary Material

XML Treatment for Clubiona jiugong
